# Sodium and magnesium in the distal convoluted tubule: no longer a couple?

**DOI:** 10.14814/phy2.13780

**Published:** 2018-07-06

**Authors:** Aylin R. Rodan

**Affiliations:** ^1^ Department of Internal Medicine Division of Nephrology and Hypertension Molecular Medicine Program University of Utah Salt Lake City Utah

The kidney matches magnesium excretion to daily intestinal magnesium absorption to maintain homeostasis of this critical divalent cation. Approximately 95–99% of magnesium filtered by the glomerulus is reabsorbed in the nephron, with 10–25% reabsorption in the proximal tubule, and 50–70% in the thick ascending limb. The last site of reabsorption is the distal convoluted tubule, where fine‐tuning of renal magnesium excretion occurs (de Baaij et al. 2015). Magnesium is reabsorbed in the distal convoluted tubule in a transcellular fashion through apically located TRPM6 (transient receptor potential melastatin type 6) (Voets et al. 2004). The sodium chloride cotransporter (NCC) is also apically localized in the distal convoluted tubule, where it reabsorbs sodium chloride. Patients with Gitelman's syndrome, who carry loss‐of‐function mutations in NCC, have hypomagnesemia (Blanchard et al. 2017), and hypertensive patients chronically treated with thiazide diuretics, which pharmacologically inhibit NCC, also develop decreased serum magnesium (Hollifield 1986). This has raised the question of whether there is functional coupling of sodium and magnesium in the distal convoluted tubule. Thiazide‐treated mice, and mice in which *Ncc* is knocked out, also have decreased serum magnesium concentrations with renal magnesium wasting and decreased expression of *Trpm6* mRNA and Trpm6 protein (Schultheis et al. 1998; Nijenhuis et al. 2005). Whether this is due to morphological changes in the distal convoluted tubule (Schultheis et al. 1998) is unclear.

To further examine the relationship between distal convoluted tubule sodium and magnesium handling, van Megen et al. examined mice with hyperactivity of distal convoluted tubule sodium reabsorption. They used a mouse model recapitulating human Gordon syndrome (also known as Familial Hyperkalemia and Hypertension or pseudohypoaldosteronism type II), in which patients have hyperkalemia and hypertension as a result of NCC overactivation due to activation of the upstream With No Lysine (WNK)/Ste20p‐related proline‐ and alanine‐rich kinase (SPAK) kinase cascade (Huang and Cheng 2015). The mice studied by van Megen et al. express a constitutively active SPAK (CA‐SPAK) mutant in the first part of the distal convoluted tubule. These mice have increased NCC expression and activity (Grimm et al. 2017). In addition, and converse to the situation with *Ncc* loss‐of‐function or chronic thiazide treatment, the CA‐SPAK mutants exhibit hypertrophy of the early distal convoluted tubule, where the mutant kinase is expressed (Grimm et al. 2017).

Functional coupling of sodium reabsorption through NCC, and magnesium reabsorption through TRPM6, would predict increased distal convoluted tubule magnesium reabsorption and hypermagnesemia in the CA‐SPAK mutant mice. In fact, plasma magnesium concentration was the same in control and CA‐SPAK mutants. The investigators, therefore, examined the response to thiazide treatment to examine NCC‐dependent magnesium transport. As previously shown (Grimm et al. 2017), increased natriuresis in response to 1 day of thiazide treatment was observed, consistent with increased NCC activity in the CA‐SPAK mice. However, thiazide‐sensitive magnesium excretion was suppressed in the mutant mice. Hence, distal convoluted tubule sodium and magnesium reabsorption do not increase in parallel in this model.

What accounts for the apparent uncoupling of distal convoluted tubule sodium and magnesium handling? A survey of transcript levels of genes involved in magnesium handling in this segment, including *Trpm6, Cnnm2, Hnf1b, Fxyd2, Slc41a1,* and *Slc41a3*, did not reveal differences, though this does not exclude posttranscriptional differences in protein expression or activity, including direct effects of SPAK on TRPM6. Another possibility is that morphological and functional changes in multiple nephron segments explain these findings. Namely, connecting tubule mass is decreased in CA‐SPAK mice, with decreased expression of epithelial sodium channel (ENaC) subunits and decreased natriuretic response to benzamil, and diminished ENaC‐dependent kaliuretic effect of short‐term thiazide treatment (Grimm et al. 2017). Magnesium excretion in response to short‐term thiazide treatment could be decreased for similar reasons, akin to the effects of the ENaC inhibitor, amiloride, which decreases renal magnesium excretion in patients with Gitelman's syndrome (Blanchard et al. 2015), and increases plasma magnesium in healthy controls treated with thiazide diuretics (Murdoch et al. 1993). TRPM6 and ENaC are both expressed in the late distal convoluted tubule (Voets et al. 2004; Nesterov et al. 2012), which is downstream of the CA‐SPAK expression site. Grimm et al. (2017) demonstrated that after 3 days of thiazide treatment, hypertrophy of the first part of the distal convoluted tubule, and atrophy of the connecting tubule, is normalized, and fractional excretion of potassium increases. Although mass of the late distal convoluted tubule did not change under any condition, the study by van Megen et al. demonstrates increased urinary magnesium excretion with more prolonged thiazide treatment, similar to the effects on potassium excretion.

The study by van Megen et al. highlights the complexity of renal magnesium handling and suggests interdependent effects of different nephron segments. Hypertrophy and increased NCC activity in the first part of the distal convoluted tubule is not sufficient to increase renal magnesium reabsorption, and 4 days of thiazide treatment, which normalizes distal tubule morphology in CA‐SPAK mutants (and, presumably, lumen‐negative potential difference due to ENaC activity), unmasks a renal magnesium wasting phenotype that is greater than that seen in thiazide‐treated control mice, despite increased *Trpm6* expression in the mutants compared with controls. This could be due to defects in the thick ascending limb, since the CA‐SPAK mutant is engineered onto a *SPAK* null background (Grimm et al. 2017). In vitro perfusion studies have demonstrated a role for SPAK in sodium transport in the thick ascending limb (Cheng et al. 2015), which is required for the generation of the lumen‐positive charge that drives paracellular magnesium transport in that segment (de Baaij et al. 2015). In this regard, the CA‐SPAK mutant mice are different from Gordon syndrome patients, in which there may be increased sodium reabsorption in the thick ascending limb (Huang and Cheng 2015; Terker et al. 2018). In the CA‐SPAK mutants, there must also be some component of proximal compensation, perhaps in the proximal tubule, to account for the normal serum magnesium at baseline. Apparently, there is more to the story than simple coupling of sodium and magnesium in the distal convoluted tubule.

## Funding Information

ARR is supported by the National Institutes of Health (DK106350 and DK110358) and the American Heart Association (16CSA28530002).

## Conflict of Interest

The author declares no conflicts of interest.
